# What Shall Appeal to Women

**Published:** 1889-01

**Authors:** 


### WHAT SHALL APPEAL TO WOMEN.

Under the above title we transfer the following excellent article from the December 1st issue of the Sanitary Era, without apology for so frequently presenting the manifold evils of "tight lacing," which for grave sanitary reasons, ought to be abandoned at once and forever.

We have no idea that any penalty whatever will deter women generally from any practice which they imagine improves their appearance or which is dictated by fashion. As we have before remarked, the only hope for riddance of that hideous deformity, the bottle-spider form, is to educate women to distinguish the graces of the human figure from those shapes that are especially created to repel us by instinctive horror from venomous creatures ; or perchance that a freak of full development may in some way seize upon the *demi-monde* of Paris, which is said to set the style for chaste and pious American ladies. If the report be true that the great ladies' tailor, Worth, refuses to fit a bottle-spider form, there is a gleam of hope for posterity in that.

The Editor of the *Medical Record* contributes a point on this subject for women, which may have some effect. Quoting Professor Lesshaft, to the effect that tight-lacing weakens the bony and muscular structures of the trunk, causing the lax and distended abdominal walls so common among women, Dr. Shrady remarks that "perhaps if young women knew that corsets which constricted the waist would eventually give them an obtrusive abdomen, they would be more careful in their lacing."

To women who admit an element of reason and conscience into their conduct in such matters, the obstructed nutrition and energy of the body and particularly of the reproductive system, process, and issue, to all generations, from constricting abdominal movements and vital currents, should appeal with decisive effect. There is nothing else in city life, we firmly believe, that is so far responsible for its proverbial decadence, and dependence on fresh blood from the country, as the extreme to which an intensified devotion to fashion impels city women in the matter of pinched waists.

A few other effects, which it may do an exceptional woman, here and there, good to read, are mentioned in the *Medical Record*, such as the following: Bouchut draws attention to the fact that pressure of stays on the intercostal spaces in tight-lacers suffering from intercostal neuralgia, may give rise to paroxysms resembling angina pectoris. Meyer states that tight-lacing decidedly manifests an injurious influence on the eye, in consequence of congestion resulting from obstructed reflux of blood from the head along the jugular veins. [This touches the feminine ruling passion.] Almost every handbook of pathological anatomy describe as peculiar, more or less deep, groove on the surface of the liver, which is very often found on the post-mortem examination of female bodies, and which is caused by tight-lacing. Professor N. Kianovsky says that the hepatic capsule adjacent to the grooves proves to be considerably thickened from chronic inflammation, while the hepatic tissue itself at such spots is dense, atrophied to more or less depth, and sometimes even void of any glandular elements. Of 1,033 necropsies at Munich, gall stones were present in men in 3.9 per cent. of the whole number, and in women in 9.9 per cent.; in as many as forty per cent. of the female cases, simultaneously with gall stones, there were found grooves and constrictions of the liver caused by stays. An important work recently published by Professor March throws a strong light on the mechanism of the formation of gall-stones in tight-lacers. Dr. W. J. Collins' experimental researches similarly elucidate pretty well why deficient bile, dyspepsia, sickness, constipation, clayey stools, headaches, chlorosis, debility, form a natural sequence of tight lacing.

Drs. Graily Hewitt, Meyers, and Helene Belts assert that tight lacing may cause displacements and flexions of the womb. Neftel's experiments showed that thoracic and abdominal compression cause anæmia. Schuter found that a lasting compression of the chest could cause albuminuria.

Kianovsky, experimenting on fourteen habitual tight lacers, found the average decrease of vital capacity of the lungs to be 357 cubic inches. The strength of inspiration and expiration was decreased also, and the respiratory excursion of the chest. Hence he concludes that the corset dooms the wearer to oxygen starvation. He also found a lowering of blood-pressure and "arterial anæmia" as a result of the lacing.

